# Commentary: Effects of breathing training on walking ability and quality of life in patients with multiple sclerosis: systematic review and meta-analysis of randomized controlled trials

**DOI:** 10.3389/fimmu.2026.1719913

**Published:** 2026-03-10

**Authors:** Yunpeng Xu, Cuifang He, Xue Han, Qiuyu Chen, Aiju Su, Hongbo Jiang, Jian Liu

**Affiliations:** 1 The First Clinical Medical College of Lanzhou University, Lanzhou, China; 2 Gansu Province Maternity and Child-Care Hospital (Gansu Provincial Central Hospital), Lanzhou, China

**Keywords:** balance, breathing training, fatigue, multiple sclerosis, quality of life, sleep, walking ability

## Introduction

Li et al. (1) published a study titled “Effects of Breathing Training on Walking Ability and Quality of Life in Patients with Multiple Sclerosis: Systematic Review and Meta-Analysis of Randomized Controlled Trials” in Frontiers in Immunology. Through comprehensive multi-database searches and rigorous screening, the authors included 21 randomized controlled trials to quantitatively synthesize the effects of breathing training on walking ability, fatigue, sleep, and quality of life. This work provides systematic evidence supporting the application of breathing training in the rehabilitation of multiple sclerosis and offers valuable insights for optimizing rehabilitation protocols and individualized interventions. Beyond its clinical implications, the study reinforces the research foundation of respiratory functional training in neurorehabilitation and provides a new perspective for understanding the mechanisms underlying functional recovery in patients with multiple sclerosis. While fully recognizing the study’s innovation and practical significance, this commentary aims to discuss several methodological and interpretative considerations.

## Unit-of-analysis bias

This meta-analysis presents a noticeable unit-of-analysis bias. In meta-analyses, when multiple intervention groups share a common control group without appropriate statistical adjustment, the assumption of independence is violated, leading to overestimation of precision, narrower confidence intervals, and an increased risk of false-positive findings (2). In the included trials, for example, Lysogorskaia et al. (3) designed three arms—yoga, physical therapy (PT), and a waiting-list control—but the review treated “yoga vs. waiting list” and “PT vs. waiting list” as two independent comparisons, thereby duplicating the same control group. Similar issues were identified in studies by Ahmadi A et al. (4), Pan Y et al. (5), Young HJ et al. (6), Oken BS et al. (7), Khadadah S et al. (8), and Heine M et al. (9), with such bias present in 8 out of 21 included trials (38.1%). In the primary outcome, the Berg Balance Scale (BBS), these studies accounted for 72.7% of the total weight, substantially compromising the reliability of the pooled results. The same concern applies to other major outcomes, such as quality of life (42.3%) and fatigue (29.8%). Notably, in subgroup analyses where fewer studies are included, the impact of this bias may become even more pronounced. According to the Cochrane Handbook, this issue can be mitigated by combining intervention groups (recommended), selecting only one comparison, splitting the control group, or employing network meta-analysis approaches (2).

## Inadequate handling of pre–post standard deviations

Most outcomes in this review were analyzed based on pre–post differences. However, the calculation of standard deviations (SDs) for these change scores lacked transparency. The authors did not report the correlation coefficient (r) assumed between pre- and post-intervention measurements, nor did they conduct sensitivity analyses to examine how varying r values might influence the results—an omission that could affect the robustness of the estimated effects. Based on the reported data compared with the original studies, it appears that a fixed r value of 0.5 may have been applied. In meta-analyses, an inappropriate choice of r can substantially affect the stability and credibility of pooled estimates. The Cochrane Handbook strongly recommends that when r is unknown, a range of plausible values (e.g., 0.25, 0.5, 0.75, or even 0.9 when necessary) be tested through sensitivity analyses to evaluate the robustness of findings, rather than relying on a single fixed value (10). The recommended calculation formula is as follows:


$$SD_{change}\sqrt{SD_{baseline}^{2}+SD_{final}^{2}-2r\cdot SD_{baseline}\cdot SD_{final}}$$

## Data and reporting inconsistencies

This review also presents several issues that may affect the robustness and credibility of its findings. First, some included studies do not fully meet the stated inclusion criteria. For instance, while this meta-analysis defined the intervention as any form of breathing training, the study by Khadadah et al. (8) employed continuous positive airway pressure (CPAP), which is a passive respiratory support therapy rather than an active breathing exercise. Its mechanism of action and rehabilitative objectives differ fundamentally from those of breathing training; therefore, it does not strictly conform to the inclusion criteria of this review. Including such studies may blur the intervention’s operational definition and introduce additional heterogeneity, thereby weakening the validity and interpretability of the pooled results. Second, inconsistencies are also observed in the numerical presentation of results. In section “3.3.4 Fatigue,” the reported meta-analytic result was SMD = -0.05; 95% CI: -0.76 to -0.24; P = 0.0001; I² = 57%. The point estimate is incongruent with the confidence interval range, making it difficult to reconcile as a coherent statistical outcome. Moreover, in **Figure 6**, the data for Heine et al., 2021 (2) show identical values for both the intervention and control groups, indicating an apparent anomaly. This study is also absent from both the reference list and **Table 1**. The only similar publication is Heine et al. (9), 2017, but upon review, the sample size does not match the values reported in this meta-analysis. These inconsistencies in data and reporting undermine the internal coherence of the review’s findings and highlight the need for greater precision in intervention definitions and transparency in data presentation in future systematic reviews.

## Conclusion

Li et al.’s study suggests that breathing training may have promising applications in improving mobility, fatigue, sleep, and quality of life in patients with multiple sclerosis. However, this commentary emphasizes that, if issues such as unit-of-analysis bias, assumptions regarding the correlation of pre–post change scores, and inconsistencies in the definition of the intervention are not adequately addressed, the pooled effect sizes and the certainty of the evidence may be substantially distorted. Therefore, the current summary estimates are better interpreted as exploratory and suggestive evidence, rather than as definitive grounds for immediate changes in clinical practice. To enhance the quality of evidence generated by future systematic reviews and meta-analyses, researchers should adhere more rigorously to the methodological standards of the Cochrane Handbook. Only within such a framework can related meta-analyses provide more reliable support for guideline development and for the design of individualized, evidence-based rehabilitation protocols for multiple sclerosis, thereby allowing promising breathing interventions to be translated into clinical practice more safely.
